# Piloting Digital Navigators to Promote Acceptance and Engagement With Digital Mental Health Apps in German Outpatient Care: Protocol for a Multicenter, Single-Group, Observational, Mixed Methods Interventional Study (DigiNavi)

**DOI:** 10.2196/67655

**Published:** 2025-09-25

**Authors:** Julian Schwarz, Kelly Chen, Hiwa Dashti, Martin Heinze, Julia Schönbeck, Darja Schubert, Benjamin Senst, Justin Speck, John Torous, Jan Wolff, Laura Uchtmann, Eva Meier-Diedrich

**Affiliations:** 1 Department of Psychiatry and Psychotherapy Immanuel Hospital Rüdersdorf Brandenburg Medical School Theodor Fontane Rüdersdorf bei Berlin Germany; 2 Faculty of Health Sciences Brandenburg Brandenburg Medical School Theodor Fontane Neuruppin Germany; 3 Division of Digital Psychiatry Beth Israel Deaconess Medical Center Harvard Medical School Boston, MA United States; 4 General Practice Dashti Eberswalde Germany; 5 General Practice Zerbaum & Colleagues, Medical Care Centre Brandenburg an der Havel Germany; 6 Peter L Reichertz Institute for Medical Informatics TU Braunschweig and Hannover Medical School Hannover Germany

**Keywords:** digital therapeutics, digital health, digital health applications, digital health app, digital health apps, digital navigator, digital navigators, digital intervention, digital interventions, eHealth, mHealth, mobile health, digital health literacy, mental health, psychiatric care

## Abstract

**Background:**

In Germany, patients often have to wait several months for psychotherapeutic treatment. Digital therapeutics (DTx) offer a promising approach for timely mental health support, but their use remains limited. Digital navigators (DNs) are specially trained medical assistants who support other health care professionals (HCPs) in selecting and using DTx. This can improve digital health literacy, increase engagement, and reduce the burden on HCPs.

**Objective:**

The DigiNavi study is the first pilot study that aims to test the feasibility of implementing DNs in general practice and outpatient psychiatric care in Germany.

**Methods:**

This mixed methods study took place at six study sites (three psychiatric outpatient clinics, three general practices) in Germany. In the prestudy, patients and HCPs participated in semistructured interviews and focus groups concerning their acceptance and expectations of DNs (phase I). The Harvard Digital Navigator Training (HDNT) was adapted, and medical assistants were trained as DNs (phase II). During the intervention, 8 patients per site (N=48) diagnosed with a mental disorder were recruited via convenience sampling and supported by DNs in using DTx for mental health for 12 weeks (phase III). Patients’ (N=48) and HCPs’ (N=18) digital health literacy, digital and technical literacy, readiness and ability to change, and clinical symptom severity were assessed before and after 12 weeks of DTx prescription and support by DNs. Patient engagement with the DiGAs (usage duration and intensity) was measured after the intervention. Quantitative data were analyzed using a pre-post design. Finally, qualitative interviews were conducted with HCPs, patients, and DNs to explore their experiences with DNs, including perceived implementation barriers.

**Results:**

The study received funding in July 2024. The prestudy including 35 participants was conducted from August to October 2024. HDNT adaptation and DN training were conducted from October to December 2024. Recruitment and quantitative baseline data collection started in December 2024, and 48 participants were enrolled by the end of March 2025. The intervention study ended in June 2025. Result dissemination and the development of strategies for the long-term implementation of DNs into the German health care system are planned until September 2025. We hypothesize that the provision of support by DNs will enhance patients’ and HCP’ digital and technical literacy, patient engagement with DiGAs, and readiness and ability to change. In addition, patients’ mental health is expected to improve after the end of the intervention.

**Conclusions:**

This is the first study to examine the feasibility and effects of DNs in German health care. The study will provide significant insights into the acceptability and feasibility of human-facilitated competency development for mental health apps in multiprofessional health care teams and their patients. The successful implementation of DNs can promote the use of DTx in Germany and thus enhance access to and the provision of health care for individuals affected by a mental disorder.

**Trial Registration:**

German Clinical Trial Register DRKS00034327; https://drks.de/search/en/trial/DRKS00034327; ClinicalTrials.gov NCT06575582; https://clinicaltrials.gov/study/NCT06575582

**International Registered Report Identifier (IRRID):**

DERR1-10.2196/67655

## Introduction

The prevalence of mental health conditions is increasing worldwide [1]. Mental health disorders, such as depression and anxiety, have a significant impact on an individual’s quality of life and, if left untreated, can become chronic [2]. This can result in a burden on health care systems and society as a whole [3]. Effective treatment typically involves psychological interventions. However, there is a significant treatment gap, particularly in low- and middle-income countries [2,4]. Lack of psychotherapy and the resulting long waiting times are structural barriers to treatment [2]. In addition, general practitioners (GPs) often do not have the resources to provide adequate treatment, even when mental disorders are recognized [5]. Personal attitudes toward psychotherapy also contribute to this gap, as many patients have a high threshold for seeking treatment and may not recognize their condition as requiring professional intervention [5].

Low-threshold digital services, such as digital therapeutics (DTx), for mental health offer scalable, evidence-based, and easily accessible solutions that can reduce waiting times and provide initial relief [6-10]. International studies indicate the great potential of digital mental health tools to improve the quality of and access to care [11]. An increasing number of countries have already introduced the possibility to prescribe DTx or financially support the purchase of mental health apps [12-14]. However, despite the growing availability of mental health apps, the use of DTx internationally remains below expectations [15,16].

The reasons for low engagement are complex [17]: A user-friendly design and interface are essential for app adoption [18]. However, a significant proportion of users lack the digital and technical literacy to use the apps intuitively and effectively [11,19]. According to a recent study, patient digital literacy represents the biggest barrier to virtual care adoption, with 71% of health care organizations identifying it as a major challenge [20]. This so-called digital divide particularly affects already underserved patient groups, such as older adult or educationally disadvantaged patients [21,22]. In addition, many patients often do not have sufficiently modern smartphones or the necessary digital health literacy to use digital mental health apps effectively [23]. However, even among populations that frequently use technology, adoption remains low. Key barriers include resistance to change and concerns about data privacy, shared by both patients and medical staff [24]. Acceptance of digital health services by health care professionals (HCPs) is an important factor influencing the adoption of digital interventions by patients [11,25]. In addition to the general concerns that HCPs may have about integrating DTx into their medical practice, they often lack the time to keep abreast of developments in DTx and how to effectively integrate them into treatment processes [7,8,24,26]. Furthermore, both HCPs and patients frequently perceive digital apps as inadequate for addressing mental health problems [23], and patients often do not adhere to the intended use and stop using the app after initial installation due to a lack of motivation [16,27]. However, research suggests that human guidance—defined as personal assistance in the selection, installation, and use of DTx—can improve both acceptance and engagement with DTx and achieve lasting positive health outcomes [28-32]. A promising approach in this context is to train selected HCPs in medical teams to become so-called digital navigators (DNs) [33-35]. The core tasks of DNs are threefold: first, to increase digital competence within psychiatric and GP treatment teams through training initiatives; second, to expand the knowledge base of HCPs and patients regarding DTx for mental health; and third, to support and accompany patients in the use of DTx. The guided use of DTx is intended to improve the care of the target group of people with mental health conditions.

In Germany, DTx are called *Digitale Gesundheitsanwendungen* (“digital health apps” [DiGAs]) and have been available since the implementation of the Digital Health Care Act (*Digitale-Versorgung-Gesetz*) in December 2019 [36]. This legislation established the framework for the approval and reimbursement of digital mental health apps. These apps must meet specific standards set by the German Federal Institute for Drugs and Medical Devices (BfArM) to ensure safety, quality, functionality, privacy, data security, and effectiveness [37]. Once approved, DiGAs can be prescribed by physicians and psychotherapists and are reimbursed by statutory health insurances. Currently, there are 72 DiGAs available, of whom 29 (40.28%) focus on mental health conditions (eg, n=7, 24.14% on depression; n=7, 24,14% on anxiety). All approved DiGAs can be found in the publicly available German DiGA directory of the BfArM [38].

Although DNs have been successfully implemented in several countries [11,32], no such role or training program currently exists in Germany. In addition, evidence from abroad cannot be easily transferred to Germany since the introduction of new roles, such as DNs, must align with existing structures, including statutory health insurance and professional licensing laws. As a result, the best practices from other countries must first be adapted to the German legal, financial, and organizational framework [39].

An exploratory pilot trial is therefore needed to gather preliminary information about the implementation of DNs in the German health care system and the feasibility of conducting a full-scale trial. The aim of this study is to (1) explore the acceptance and expectations of HCPs and patients in Germany toward DNs (2); adapt the Harvard Digital Navigator Training (HDNT) for the German health care system; (3) evaluate the trial implementation of DNs, including their impact on patients and other HCPs; and (4) investigate options for perpetuating DNs in the German health care system.

## Methods

### Study Setting

This study protocol was developed in accordance with SPIRIT (Standard Protocol Items: Recommendations for Interventional Trials) guidelines [40]. The SPIRIT checklist was used to ensure completeness and transparency in the reporting of key methodological aspects (see Multimedia Appendix 1 for the SPIRIT checklist).

Within the framework of the DigiNavi study (duration: July 2024-September 2025), DNs will be piloted in GP practices (n=3) and psychiatric outpatient clinics (n=3) in different rural and urban regions of the federal state of Brandenburg, Germany, based on the DN model of the Harvard Medical School [41,42]. Specifically, study centers are located in the Brandenburg districts of Märkisch-Oderland, Oder-Spree, Barnim, Brandenburg an der Havel, and Dahme-Spreewald. Study sites were selected to ensure proximity between a primary care practice and a psychiatric outpatient clinic to facilitate networking.

### Role of Digital Navigators

**Figure 1:**
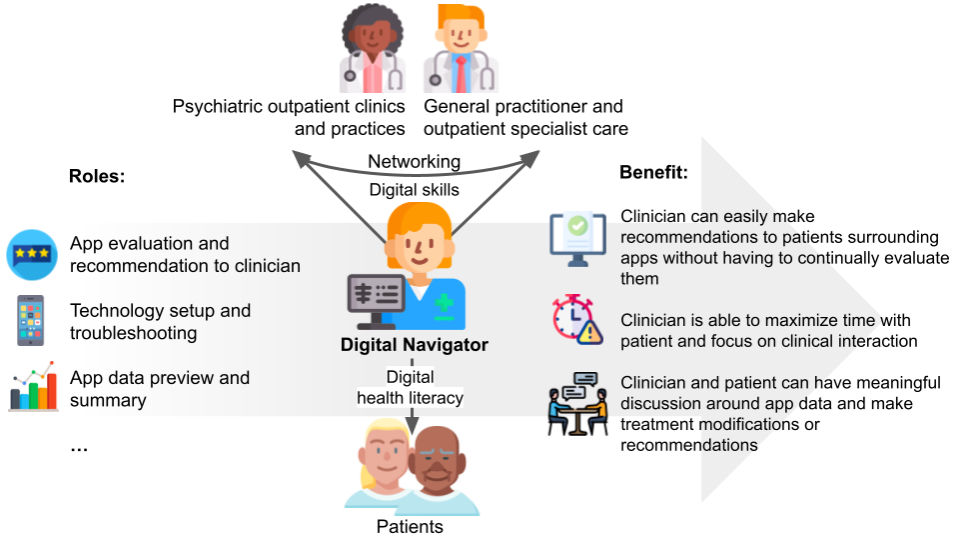
Role and tasks of DNs adapted from Wisniewski and Torous [34]. DN: digital navigator.

According to Perret et al [11], DNs have at least three distinct roles (see Figure 1): First, it is their task to keep their knowledge of available mental health apps up to date and to evaluate these apps based on the available evidence. For this purpose, DNs can refer to the MindApps database of Harvard University. In this way, DNs can advise HCPs on the selection of suitable health apps and provide ongoing assistance to patients in using the app. Second, DNs are responsible for teaching the multiprofessional care team the technical and digital basics and skills, as well as informing team members about available mental health apps and their specific features. This requires constant availability to team members and patients in the case of questions or problems (troubleshooting). Third, it is the DNs’ responsibility to process the data generated by patients’ use of the mental health apps and prepare the data for the HCPs in a way that is useful for treatment.

### Complex Health Care Intervention

**Figure 2:**
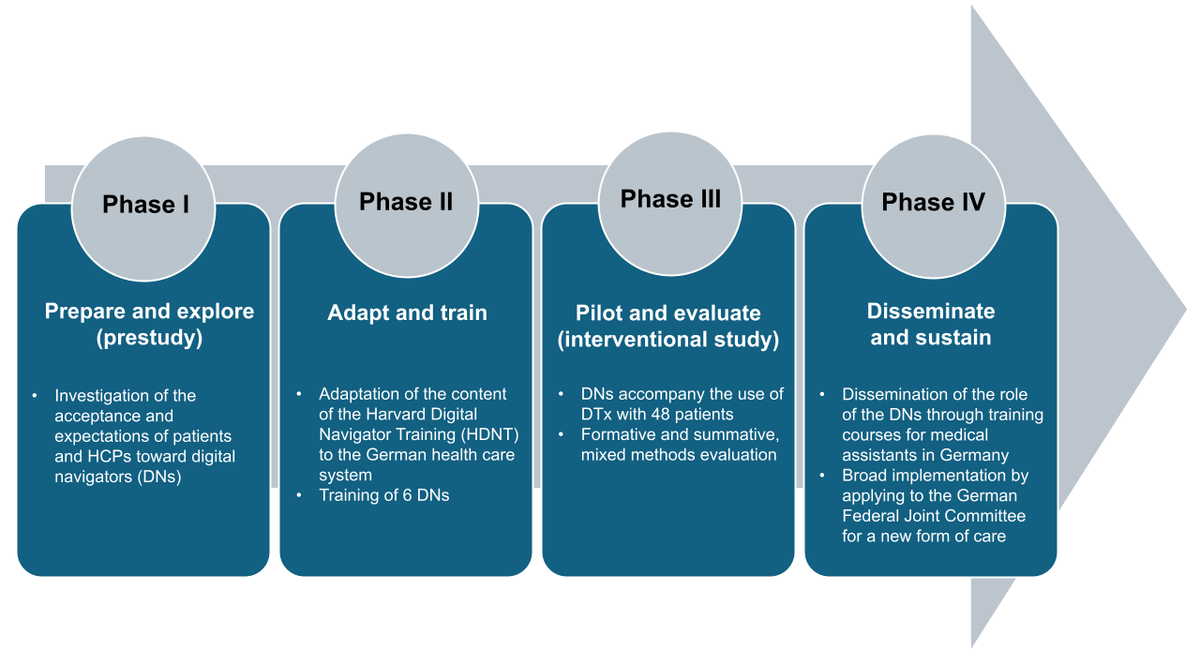
Research phases of the DigiNavi study. DN: digital navigator; DTx: digital therapeutics; HCP: health care professional; HDNT: Harvard Digital Navigator Training.

The methodological implementation is strictly based on the UK Medical Research Council (MRC) guidelines for the evaluation of complex interventions [43]. A complex intervention is defined as a health care intervention or program that consists of a number of interacting components, each of which may contribute to the overall outcome in different ways [44]. The impact of the intervention typically depends on the interaction between these different components. The use of DNs in multiprofessional care teams is a complex intervention with several interacting components, as the role of DNs comprises several components in terms of content (see Figure 2) and requires evaluation at multiple levels. In this regard, its evaluation must include questions about digital health literacy, implementation conditions, the role and function of DNs, treatment processes, and impact factors, in addition to clinical endpoints. Furthermore, the evaluation process should consider the perspectives of patients, HCPs, and DNs themselves.

### Process Evaluation

To evaluate complex interventions, such as the pilot of DNs, and to gain insight into the impact of the intervention in practice, elements of process evaluation were incorporated into the study design [44]. In this sense, the design of this study follows the fundamental recommendations of the UK MRC for process evaluation [43]. Accordingly, the intervention, any prior assumptions, contextual factors, implementation processes, influencing factors, and outcomes were carefully observed, analyzed, and described in order to facilitate an informed interpretation of the study results [43]. A mixed methods approach was adopted, using both quantitative and qualitative methods for data collection and analysis to provide a comprehensive picture of the implementation of the intervention. In our analysis, we will integrate quantitative and qualitative data to examine, for instance, how different local implementations are related to the implementation experiences.

### Study Population

#### Recruitment

Both patients and HCPs (GPs, outpatient psychologists and psychiatrists, medical assistants) collaborated with DNs and were therefore considered as relevant stakeholders and included in this study.

HCPs and DNs (usually medical assistants at each study site) were recruited by the study team. In addition, raters (n=6, typically psychologists) were assigned to each study site to conduct data collection. Future DNs and raters were approached by members of the study team and informed about the study, as well as the compensation for their work. DN candidates and raters were then employed as part of the study team.

Patients were approached by their HCPs and informed about the DigiNavi study, including their compensation. Interested patients received further written and verbal information from the respective DNs at the study site and were enrolled into the study upon providing written informed consent. New participating patients were recruited in each phase of the study, and the pool of previously recruited participants was used in subsequent phases. For the intervention study, each center screened subjects until the target population was achieved (n=8 patients/site).

#### Sampling

A report on DiGAs by the German Association of Statutory Health Insurance Funds (GKV-Spitzenverband) indicates that in Germany in 2023, users of DiGAs for mental health conditions were on average 43 years old and 70% female [45]. Internationally, sociodemographic factors appear to be similar, as shown by a study examining diversity in clinical trials of DTx, which found that the median reported mean age across studies is 43.3 years, with 65% of participants being female [46]. To better address the specific needs of younger, older, and male patients, convenience sampling with a balanced gender ratio was used for all patient-related surveys conducted as part of this study. In addition, marginalized groups in the German health care system were prioritized for recruitment to address inequalities in mental health care through the use of DNs [47]. This included the selection of at least 1 individual who was non-White (n≥1); nonnative German-speaking (n≥1); lesbian, gay, bisexual, transgender, queer, questioning, intersex, asexual, and other identities (LGBTQIA+; n≥1); and older than >65 years (n≥1) [48] for study participation. The sample size for each study phase was not derived statistically but determined based on feasibility considerations. As this is a pilot study with an exploratory character, the primary objective is not to achieve statistical power for hypothesis testing but to gather preliminary data on the intervention’s implementation, acceptability, and potential effects. Given the focus on the practical implementation of the intervention in a real-world setting, nonrandomized allocation using a convenience sample was chosen for the intervention group.

### Eligibility Criteria for Patients

Patients provided written informed consent before any study procedures occurred (see Multimedia Appendix 2 for a sample informed consent form). Patients eligible for the trial had to comply with all the following inclusion and exclusion criteria listed in Textbox 1.

**Inclusion and exclusion criteria for this study.**

Inclusion criteria:

- Diagnosis according to the
*International Classification of Diseases and Related Health Problems, 10th Revision*
  (ICD-10) codes F01-F06.x or F06.7
- Ability to provide informed consent
- Minimum age of 18 years
- Absence of significant cognitive deficits, as indicated by severe organic brain disease
- Sufficient language skills to complete an interview in German
- Sufficient modern hardware (eg, smartphone, tablet, or personal computer) available
- Basic knowledge of how to use a smartphone

Exclusion criteria:

- Ongoing or recent (≤3 weeks) qualified psychiatric or psychotherapeutic treatment
- Acute danger to self or others
- Disturbance of orientation or reference to reality
- Diagnosis of intellectual impairment

### Eligibility Criteria for Health Care Practitioners

Participating HCPs must be at least 18 years of age and provide informed consent to participate. HCPs must be either GPs or psychologists or physicians employed in psychiatric centers. We therefore assumed basic psychological expertise in order to diagnose mental disorders. DNs are HCPs who were recruited from resident medical assistants at the study centres. In Germany, these occupational groups often work as receptionists, who usually work at the registration desk of health care facilities and perform mainly administrative and partly nursing tasks (eg, administering depot medication or carrying out treatments ordered by a medical practitioner). The medical assistants know the patients well and work closely with other HCPs, which predestines them to take on the role of a DN.

### Study Design

The DigiNavi study is designed as a multicenter, single-group, observational, mixed methods interventional study, incorporating elements of participatory design. This study consists of four research phases (see Figure 2): a prestudy (phase I), training of DNs (phase II), a 12-week intervention where DNs support the use of DiGAs (phase III), and steps toward integrating the DN role into the German health care system (phase IV). The following research questions will be addressed in the four phases:

- Phase I: What are the levels of acceptance and expectations of HCPs and patients toward DNs?
- Phase II: What responsibilities and specific skills should DNs have in the German health care system? How can these findings contribute to the need-based introduction of this new role?
- Phase III: What impact does the advice and support provided by DNs have on the digital health literacy of patients and HCPs? What impact does the increased integration of DiGAs into treatment have on their use and the therapeutic relationship between patients and HCPs? What are the challenges and barriers that arise when piloting DNs?
- Phase IV: What measures are necessary to ensure the long-term implementation of DNs in the German health care system?

### Data Collection and Analysis

The following sections provide a concise overview of the data collection procedures (primary and secondary measures). All primary and secondary outcomes will be assessed before and after the 12-week intervention (pre- and postassessment). For an overview of enrollments, interventions, and assessments, see Multimedia Appendix 3. The subsequent sections offer a comprehensive description of the specific data collection procedures that were implemented in each phase of the study.

#### Primary Outcome Measures

One primary outcome measure will be the impact of DNs on patients’ and HCPs’ digital health literacy. Digital health literacy will be assessed with two validated instruments, the Revised German eHealth Literacy Scale (GR-eHEALS) [49,50] and the Digital Health Literacy Index (DHLI) [51], which can already detect changes in smaller samples [52], before and after the 12-week intervention. The eHealth Literacy Scale (eHEALS) is the most widely used international questionnaire for the assessment of digital health literacy. It is a self-assessment tool that focuses on digital information–seeking skills [50]. We used the GR-eHEALS instead of the original eHEALS to ensure linguistic and cultural appropriateness for our German-speaking target group, since it is an adapted and validated version that considers language nuances and health care system specifics relevant to Germany [49]. The DHLI complements the GR-eHEALS in assessing interactive skills, such as the use of (web-based) health apps [51]. Further important primary outcome measures were the digital and technical literacy of patients and HCPs, their readiness and ability to change, as well as patients’ engagement with DiGAs. The following self-assessment instruments were used for this purpose: the information and communication technology (ICT) Self-Concept Scale [53], the Brief Scale of Technological Readiness [54], and the Digital Use survey from Harvard University and the Readiness for Change survey [55]. Engagement with DiGAs was assessed via structured interviews conducted by the study team.

#### Secondary Outcome Measures

As a secondary outcome measure, the severity of the patient’s disease was assessed and compared before and after the intervention using validated scales. This measure allowed for a comprehensive evaluation of the symptom burden, functional status, and overall clinical improvement.

The Global Assessment of Functioning (GAF) [56] and the Clinical Global Impression (CGI) [57] scales are brief clinician-rated tools that capture the clinician’s view of the patient’s overall functioning and treatment response and are widely used to evaluate symptom severity and improvement. To assess specific symptom domains, the following instruments were used: the Hamilton Anxiety Rating Scale (HAM-A) [58], which is an instrument that measures the severity of anxiety symptoms, and the Hamilton Depression Rating Scale (HDRS) [59], which evaluates depressive symptomatology across multiple dimensions, including mood, sleep, and somatic complaints. These clinician ratings were complemented by the Beck Depression Inventory-II (BDI-II) [60], a widely used self-report questionnaire that captures the patient’s subjective experience of depressive symptoms. Additionally, sleep quality was assessed using the Insomnia Severity Index (ISI) [61], a brief self-report measure that evaluates the nature, severity, and impact of insomnia, which often co-occurs with mental health conditions and may influence treatment outcomes.

Table 1 provides an overview of the instruments used for data collection and analysis. The specific methodological details for each study phase are described in the following sections.

**Table 1:** Construct and data collection used for pre- and postassessment of the interventional study.

Outcome and participants		Construct	Data collection instrument
**Primary outcomes**			
	Patients and staff	Digital health literacy	DHLI^a^ (online self-assessment survey) GR-eHEALS^b^ (online self-assessment survey)
	Patients and staff	Digital and technical literacy	ICT^c^ Self-Concept Scale (online self-assessment survey) Brief Scale of Technological Readiness (online self-assessment survey) Digital Use survey (online self-assessment)
	Patients and staff	Readiness and ability to change	Readiness for Change survey (online self-assessment)
	Patients	Engagement	Duration and frequency of DiGA use (observer rating)
**Secondary outcomes**			
	Patients	Severity of disease	GAF^d^ (observer rating) CGI^e^ (observer rating) HAM-A^f^ (observer rating) HDRS^g^ (observer rating) BDI-II^h^ (online self-assessment survey) ISI^i^ (online self-assessment survey)

^a^DHLI: Digital Health Literacy Index.

^b^GR-eHEALS: Revised German eHealth Literacy Scale.

^c^ICT: information and communication technology.

^d^GAF: Global Assessment of Functioning.

^e^CGI: Clinical Global Impression.

^f^HAM-A: Hamilton Anxiety Rating Scale.

^g^HDRS: Hamilton Depression Rating Scale.

^h^BDI-II: Beck Depression Inventory-II.

^i^ISI: Insomnia Severity Index.

### Research Phases

#### Phase I: Prepare and Explore (Prestudy)

A first exploratory prestudy examined the acceptance of and expectations of DNs from the perspective of patients and HCPs. This qualitative study involved 7 GPs, 11 outpatient psychiatrists/psychologists, and 17 of their patients from the service areas of the study sites. Specifically, 17 semistructured interviews (n=4, 57.1%, GPs; n=4, 36.4%, outpatient psychiatrists/psychologists; and n=9, 52.9%, patients) and 4 focus groups were conducted. Two focus groups with patients (n=8, 47.1%), one focus group with GPs (n=5, 71.4%), and one focus group with outpatient psychiatrists/psychologists (n=7, 63.6%) were conducted. The interview guidelines were developed based on knowledge gaps identified through an extensive literature review. The questions related to what opportunities and barriers patients and professionals saw in the implementation of DNs, how it might change the HCP-patient relationship, and what they think should be considered when introducing DNs (see Multimedia Appendix 4 for the prestudy interview guideline for patients). In addition, the sociodemographic characteristics of the participants were collected [62].

The developed survey instruments and interview guides were pretested before implementation. As part of the pretest, one interview was conducted with a patient and one with an HCP. The collected qualitative data were transcribed, pseudonymized, and analyzed using MAXQDA software following a thematic analysis to elaborate stakeholders’ acceptance and expectations of DNs [63].

#### Phase II: Adapt and Train

##### German Version of the Harvard Digital Navigator Training

Three researchers from the study team participated in a multiday training and coaching program on the implementation of DNs during field visits at Harvard Medical School [64]. Furthermore, the content of the certified HDNT was translated into German and adapted to the German context based on the findings from the focus groups and interviews. Depending on the results of the qualitative data (eg, information about the skills that are particularly important for DNs), the HDNT was adapted. If necessary, new modules were developed and added. Additionally, there was a participatory process in terms of a modified Delphi study [65] that involved patients (N=9) and HCPs (N=17) in discussion groups at three time points, following the levels of the European Commission’s Digital Competence Framework for Citizens (DigComp 2.0) [66]. During these discussion groups, selected components of the HDNT were presented and evaluated for their suitability in the German context. The content of the conversation was documented by a member of the study team in the form of field notes, which were subsequently presented to the entire study team and discussed within the team. Based on these insights, the study team developed an adapted version of the HDNT and designed the training sessions for the German DNs.

##### Training the Digital Navigators

Subsequently, clinical staff (preferably medical assistants who participated in phase II; n=6, 35.3%) were trained with the adapted HDNT as DNs in three training sessions. Each session lasted for 3 hours and took place over 3 consecutive weeks. The first session was held in person at the study site in Rüdersdorf. The following two sessions were web based. According to the predefined roles of DNs, the training entailed general knowledge about existing DiGAs and how to keep the knowledge of available DiGAs up to date. The DNs were provided with a study smartphone and a test account for each of the mental health DiGAs available. As part of the training sessions, all DiGAs available at the time of training were introduced and tested together, ensuring that all DNs could become familiar with the DiGAs, both theoretically and practically. This process enabled the DNs to gain a comprehensive overview of all available DiGAs, thus allowing them to recommend the most suitable one later. Additionally, technical issues and difficulties could be identified and resolved immediately.

Beyond the knowledge about the DiGAs, the DNs also received mental disorder–specific training. Currently, DiGAs are available for the following mental illnesses: anxiety disorders, depression, and eating disorders. The DNs were provided with an overview of the diagnosis, prevalence, and treatment of these mental illnesses. The main sources were the *International Classification of Diseases and Related Health Problems, 10th Revision* (ICD-10) [67] for diagnosis and the Association of Scientific Medical Societies (AWMF) level S3 treatment guidelines for mental illnesses [68]. This contributed to raising awareness and knowledge of mental health disorders, particularly among DNs working in GP settings, enabling them to assess these disorders competently.

Furthermore, DNs were instructed on how to deliver training on DiGAs to their multiprofessional teams in their workplace and how to use and process data generated by patients by using the DiGAs. Additional training content entailed recruitment of participants, technological troubleshooting, or the conduction of motivational check-in appointments with participants.

A key reference for DNs was the DIGAnavigator website, which we will develop based on the model of MindApps, the platform created by Harvard University. This new website will focus on integrating and evaluating German DiGAs. Initially, all DiGAs for mental health approved by the BfArM [38] in Germany will be listed on the website. In addition, detailed psychoeducational content on the use of DiGAs for mental health conditions will be developed and incorporated into the website.

##### Training the Raters

In addition, 6 psychologists were trained as raters to conduct participant recruitment and data collection. The training session was conducted at the study site in Rüdersdorf and lasted 3 hours. During the training session, the raters learned more about the data collection process in the study. Specifically, they learned which instruments are used for pre- and postassessment, how data for self-assessment are collected, and what to consider when conducting an interview about the patients’ severity of disease. This training aimed to standardize testing and to promote consistency in data collection.

#### Phase III: Pilot and Evaluate (Interventional Study)

In the intervention phase, the trained DNs trained the multiprofessional teams at the six study sites to select and integrate DiGAs for mental health into the treatment of 48 patients (n=8, 16.7%, per site). The DNs provided at least one training session for their teams. A guideline for this training was provided by the study team. Based on the findings from phases I and II, the content and structure of the intervention were finalized. It was established that selected patients would use a DiGA for 12 weeks and receive guidance and support from trained DNs during these 12 weeks, encouraging regular use of a DiGA tailored to their individual needs. To enhance adherence, participants were regularly asked about any challenges they encountered during check-up appointments with their DNs. To promote engagement, DNs suggested practical strategies, such as integrating DiGA use into daily routines or identifying suitable time frames for engagement. The study team supported the DNs through monthly web-based meetings. In these meetings, DNs received up-to-date information about the study progress, got an opportunity to ask questions, and exchanged experiences. During the intervention phase, DNs contacted the study team at any time if they had questions or encountered any issues. In addition, process data, such as the number and duration of contacts between patients and DNs, were collected during the intervention to enable the identification and validation of any qualitative differences in implementation at the different study sites.

The trained raters (n=6) conducted data collection with patients (N=48) and HCPs (N=18; n=3, 16.7%, per site) at each study site at two timepoints: before the 12-week intervention (preassessment), and directly after completing the intervention (postassessment). The end of the intervention phase was defined as the date of DiGA activation plus a 3-month DN support period. The study team also met with the raters once a month in a web-based meeting to discuss the progress of the preassessments and later postassessments. During these meetings, raters got an opportunity to ask questions and receive assistance with any issues or challenges they may have encountered.

All patients and HCPs received an online self-assessment form (administered by the online survey service LimeSurvey), assessing sociodemographic parameters (eg, age, gender), digital health literacy, digital and technical literacy, readiness and ability to change, and severity of disease (see Table 1). Additionally, patients were interviewed on the severity of their disease and app engagement.

The collected sociodemographic characteristics were analyzed using descriptive statistics and presented in an appropriate format, such as age distribution and gender ratio. Quantitative primary data collected (eg digital health literacy, severity of disease) were analyzed using descriptive statistics in a pre-post comparison using paired *t*-tests or Wilcoxon signed-rank tests, depending on the distribution of the data. Effect sizes were reported to assess the magnitude of changes, and multiple linear regression models were used to control for potential confounders. Analyses were performed using SPSS with a statistical significance set at α=.05.

After the intervention, the study team conducted qualitative semistructured interviews and focus groups with 25 participants in total (n=13, 52%, patients; n=6, 12.5%, DNs; and n=6, 12.5%, HCPs) to assess the opportunities and implementation barriers to implementing DNs, as well as the effects of counseling and support by the DNs. The collected qualitative data were transcribed, pseudonymized, and analyzed using qualitative content analysis according to Kuckartz and Rädiker [69]. The diverse and complex narratives of the participants were recorded and explored using a multistage approach. The formation of categories and subcategories enabled the content of the information to be structured. The iterative procedure increased the comprehensibility of the information generated but also allowed flexibility and an interpretative approach. To ensure high-quality reporting, the COREQ (Consolidated Criteria for Reporting Qualitative Research) checklist (Multimedia Appendix 5) was followed [70].

#### Phase IV: Disseminate and Sustain

This study phase includes the dissemination of the study results, as well as the development of strategies to ensure the long-term integration of the pilot innovation into the German health care system. This includes the continuation of the pilot project beyond the end of the study by maintaining the DiGAnavigator website, training medical assistants at the Federal State of Brandenburg’s Medical Association (LAEKB), and preparing the accreditation of the training program for medical assistants to become DNs through the LAEKB. Preliminary scenarios for the remuneration of DN activities are currently being formulated. Results of the study will be disseminated regardless of the magnitude or direction of the effect.

### Ethical Considerations

The study was approved by the Ethics Committee of the Brandenburg Medical School (MHB) (reference number: 218062024-BO-E). The study was registered at the German Clinical Trials Registry (DRKS00034327) and at ClinicalTrials.gov (NCT06575582). The DNs or HCPs introduced the study to patients. Patients also received information sheets. The DNs or HCPs discussed the trial with patients in the light of the information provided in the information sheets. All participants were informed about the possibility to withdraw their consent at any given time. Afterward, the DNs or HCPs obtained written informed consent from the participants of the study. If a patient chose to discontinue during the intervention phase, the DN or HCP conducted an informative discussion to explore the underlying reasons in order to understand potential barriers to the use of digital mental health interventions. For their participation in either the preliminary study or the intervention study, patients received 25 euros (US $23.92).

All self-assessments were conducted using LimeSurvey, a web-based platform. This approach reduced the risk of data loss or unauthorized access, as no local storage on individual devices was required. It also allowed the study team to monitor data entries in real-time, enabling timely detection of incomplete or erroneous submissions. If necessary, raters could be informed so they could follow up with participants.

To ensure data protection, each participant was assigned a pseudonymized study code that does not allow identification. Qualitative data, such as transcripts from focus groups and interviews, were securely stored on the study team’s local, password-protected computers. In accordance with data protection regulations, all data collected were pseudonymized to safeguard participant information and will be deleted after 10 years, ensuring compliance with legal and ethical requirements, while allowing sufficient time for scientific evaluation.

## Results

The study received funding in July 2024. The prestudy, including the collection of qualitative data, was conducted from August to October 2024. The adaptation of the HDNT and the training of the DNs took place from October to December 2024. Data analysis was completed in March 2025, and the data are being prepared for publication. Recruitment of participants and collection of quantitative data (preassessment) commenced in December 2024 and continued through March 2025. By the end of March 2025, a total of 48 participants had been enrolled. The intervention study, in which participants used DiGAs and received support from the DNs, started in December 2024 and ended in June 2025. The second collection of quantitative data (postassessment) began once the first participant completed the intervention phase (March 2025) and continued until early June 2025. The qualitative data collection was conducted in June 2025.

Dissemination of results and the development of strategies for the long-term integration of this pilot innovation into the German health care system have been planned between March and September 2025. The study will provide important insights into the acceptability and feasibility of human-facilitated competency development for mental health apps in multiprofessional health care teams and their patients.

## Discussion

### Summary

International studies indicate the great potential of digital mental health tools to improve the quality of and access to care [11]. However, the actual use and integration of these apps is still limited, even in an international context, partly due to a lack of relevant technical and digital literacy among HCPs and patients alike [34]. DNs have the potential to mitigate these barriers, promote digital health literacy, and ultimately lead to improved care outcomes [11,33].

The study will provide important insights into the acceptance of DNs by HCPs and patients (phase I), which, in combination with the participatory adaption and piloting of the German DN training, will contribute to a target-oriented implementation in the pilot region (phase II) and beyond. The open access publication of the adapted and field-tested manual and all training materials for the DN training (including the MindApps database) in German will facilitate the independent implementation of the innovation by outpatient care providers and enable its extension to other disciplines and settings (eg, day clinics or even inpatient care). To ensure a broad implementation and continuation of the innovation, the project team is preparing the accreditation of the training program for medical assistants to become DNs by the LAEKB. At the end of the project period, a roll-out phase is planned, during which the innovation will be transferred to the member practices of the Brandenburg General Practitioners Association.

The role of DNs in multiprofessional medical teams is a novel approach within the German health care system, which is why it has not yet been researched. The DigiNavi study will adapt, pilot, and evaluate the role of DNs in a participatory manner for the German health care system. The planned study will provide valuable insights into the acceptance, expectations, challenges, and opportunities associated with the role of DNs in improving health care.

### Limitations

This study is limited by the size and composition of the sample, which was selected based on convenience rather than random sampling. Due to the small sample size, the study is not powered to detect statistically significant effects. However, it reveals trends of potential changes that should be further explored and tested in future randomized studies. Furthermore, the small sample size increases the risk of type II errors and limits the generalizability and transferability of quantitative findings. The nonrandomized sampling constrains the ability to draw causal inferences, and the findings cannot be generalized to the entire population of patients with mental health conditions in Germany. Although the study is designed to assess feasibility, not effectiveness, we recommend that future research include a randomized controlled design to evaluate causal effects more robustly.

Moreover, patients treated in psychiatric outpatient clinics often have more severe mental health conditions compared to those in general outpatient psychotherapy, further limiting the generalizability of the results. In addition, the focus on patients with mental health conditions is a limitation in itself, so the results cannot be generalized to patients with somatic conditions or to individuals without illnesses. Furthermore, it can be assumed that the study will attract a larger number of technologically proficient HCPs and patients (self-selection). This may result in overrepresentation of this particular patient and HCP group.

Additional limitations arise from the outpatient setting of the study. In inpatient care, the ability to prescribe DiGAs is restricted. These contextual conditions, in combination with the exploratory nature of the pilot study, do not allow for the integration of these aspects into the present investigation.

In addition, the study is being conducted without a comparison group (ie, with only one condition). This makes it difficult to identify and control for confounding factors and to attribute observed changes, such as in digital literacy or psychological health, clearly to the intervention. However, the primary goal of the DigiNavi study is to investigate the feasibility of the innovation, not to provide evidence of its effectiveness. The sample size and composition chosen are therefore justified by the exploratory nature of the planned study and are appropriate for a pilot project. The implementation of a larger randomized controlled trial may be a future goal for research efforts to assess effectiveness and causal relationships.

Furthermore, the limited funding period of only 14 months has resulted in the intervention phase being constrained to a duration of 3 months per patient. As a result, the study design does not include a follow-up component, precluding any assertions regarding the evolution of the outcomes of interest over a 6- or 12-month period.

Further studies are needed to test DNs in inpatient and outpatient settings, or in the somatic field, to determine whether the hypothesized effects of DNs can be confirmed in other settings and patient groups and whether DNs can be established as a sustainable tool for improving the care of patients with mental health conditions. In addition, further international studies involving different health care systems would be of great value. The findings of this study, together with the easily accessible training materials on the project website [71], should encourage and facilitate further research.

## Appendix

Multimedia Appendix 1 SPIRIT (Standard Protocol Items: Recommendations for Interventional Trials) checklist.

Multimedia Appendix 2 Sample informed consent form.

Multimedia Appendix 3 Schedule of enrollments, interventions, and assessments. DN: digital navigator; HDNT: Harvard Digital Navigator Training.

Multimedia Appendix 4 Prestudy interview guideline for patients.

Multimedia Appendix 5 COREQ (Consolidated Criteria for Reporting Qualitative Research) checklist.

## Abbreviations

BDI-II: Beck Depression Inventory-II
BfArM: German Federal Institute for Drugs and Medical Devices
CGI: Clinical Global Impression
DHLI: Digital Health Literacy Index
DiGA: digital health app
DN: digital navigator
DTx: digital therapeutics
eHEALS: eHealth Literacy Scale
GAF: Global Assessment of Functioning
GP: general practitioner
GR-eHEALS: Revised German eHealth Literacy Scale
HAM-A: Hamilton Anxiety Rating Scale
HCP: health care professional
HDNT: Harvard Digital Navigator Training
HDRS: Hamilton Depression Rating Scale
ICD-10: International Classification of Diseases and Related Health Problems, 10th Revision
ICT: information and communication technology
ISI: Insomnia Severity Index
LAEKB: Federal State of Brandenburg’s Medical Association
MRC: Medical Research Council

## Data Availability

The datasets generated or analyzed during this study will not be publicly available but will be available from the corresponding author upon reasonable request.
