# Supplementary material for: Piloting Digital Navigators to Promote Acceptance and Engagement With Digital Mental Health Apps in German Outpatient Care: Protocol for a Multicenter, Single-Group, Observational, Mixed Methods Interventional Study (DigiNavi)
Source: JMIR Res Protoc. 2025 Sep 25;14:e67655. doi: 10.2196/67655 (PMC12511820; doi:10.2196/67655)
Supplement: Multimedia Appendix 2 [file resprot_v14i1e67655_app2.pdf]

Gefördert durch:

Bundesministerium
für Gesundheit

aufgrund eines Beschlusses
des Deutschen Bundestages

MEDIZINISCHE
HOCHSCHULE
BRANDENBURG

Declaration of consent for participation in the study

"Digital Navigators for Acceptance and Competence Development with Mental Health Apps: Interventional Study with a Mixed-Method Process Evaluation" (DigiNavi)

Funding: Supported by funds from the Federal Ministry of Health based on a resolution of the German Bundestag; announcement "Pilot project for the implementation and consolidation of digital health approaches in healthcare, rehabilitation and nursing" dated 24.07.2023
Funding reference: 2524FEP10A

family name: ______________________

first name: _________________

date of birth: ___________________

I am participating in the DigiNavi study as:

- patient
- digital navigator
- general practitioner
- other practitioner / other practitioner
- other namely: _______________

I have been fully informed about the nature, significance and scope of the planned survey as part of the study on the introduction and investigation of digital navigators. I have read and understood the information sheet. I have also been informed verbally in detail.

I have been made aware that participation in the above-mentioned study can be cancelled by either party at any time without giving reasons and without any disadvantages for me. I understand that personal data is collected in scientific studies. The forwarding, storage and evaluation of this study-related data is carried out in accordance with legal regulations and requires my voluntary consent.

I agree that my data collected as part of the above-mentioned study may be recorded on electronic data carriers, audio recordings made and passed on in encrypted form to the responsible researchers of the DigiNavi study group for scientific evaluation without my name being mentioned.

I agree that the data collected may be passed on to external third parties for processing the following doctoral project: ‘Acceptance and expectations towards digital navigators from the perspective of patients and practitioners: Qualitative study’.

I consent to this data being analysed and stored on a computer not connected to a network separately from all other study data for the purposes of the study.

If it should be necessary for the research project to link my data collected as part of the DigiNavi study with my other personal data, this will only be done with my express consent.

I agree that the study team may contact me after the survey has been completed if necessary.

All data will be deleted in accordance with the ten-year verification obligation of the German Research Foundation and the legal requirements.

I understand that participation in the above-mentioned study is voluntary and can be cancelled by me at any time. I understand that I can withdraw completely at any time without giving any further reasons and without incurring any disadvantages.

I have had sufficient time and opportunity to ask open questions about the purpose, procedure and background of the study and have received an information letter about the study.

The following is only valid for participating patients: I am aware that by signing this consent form, I release my healthcare professional from his/her duty of confidentiality towards the study team.

family name, first name of digital navigator: ________________________________

name of the practice/outpatient psychiatric clinic: ___________________________

I voluntarily agree to participate in the above-mentioned study.

____

date family and frist name signature study participant

I confirm that I have verbally informed the study participant about the above-mentioned study and have given him/her written information about it.

____

date family and first name signature study assistant

Contact details of study assistant at your study center:

name of study center:

address of the study center:

family name, first name of the study assistant:

telephone number of study assistant:

e-mail address of study assistant:

Contact details of the study management:

Dr. Julian Schwarz (julian.schwarz@mhb-fontane.de)
Immanuel Albertinen Diakonie gGmbH
Klinik für Psychiatrie und Psychotherapie
Zentrum für Seelische Gesundheit,
Immanuel Klinik Rüdersdorf
Universitätsklinikum der Medizinischen Hochschule Brandenburg
Seebad 82/83, 15562 Rüdersdorf
