# Supplementary material for: Piloting Digital Navigators to Promote Acceptance and Engagement With Digital Mental Health Apps in German Outpatient Care: Protocol for a Multicenter, Single-Group, Observational, Mixed Methods Interventional Study (DigiNavi)
Source: JMIR Res Protoc. 2025 Sep 25;14:e67655. doi: 10.2196/67655 (PMC12511820; doi:10.2196/67655)
Supplement: Multimedia Appendix 3 [file resprot_v14i1e67655_app3.pdf]

	STUDY PERIOD						
	Enrolment	Pre-Study (Phase I)	Training (Phase II)	Intervention (Phase III)			Disseminate (Phase IV)
**TIMEPOINT**	**$-t_1$**	**t_0**	**t_1**	**t_2** *(pre-test)*	**t_3**	**t_4** *(post-test)*	**t_5**
**ENROLMENT:**							
**Eligibility screen**	X						
**Informed consent**	X						
**Allocation**		X					
**INTERVENTIONS:**							
Adaption of HDNT			X				
DN Training			X				
Rater Training			X				
Support by DNs				◆—	—	—◆	
Application for accreditation of new training programme							X
**ASSESSMENTS:**							
Acceptance and expectations toward DNs		X				X	
Severity of disease				X		X	
Digital health literacy				X		X	
Digital and technical literacy				X		X	
Readiness to change				X		X	
